# Motivation in caregiving among mothers of children with intellectual and developmental disabilities in Iran: A qualitative study

**DOI:** 10.1186/s12887-024-04957-y

**Published:** 2024-07-25

**Authors:** Seyed Javad Hosseini, Monir Ramezani, Farah Ashrafzadeh, Jamshid Jamali

**Affiliations:** 1 Department of Pediatrics, School of Nursing and Midwifery, Mashhad University of Medical Sciences, Mashhad, Iran; 2 Nursing and Midwifery Care Research Center, Mashhad University of Medical Sciences, Mashhad, Iran; 3 Department of Pediatrics, School of Medicine, Mashhad University of Medical Sciences, Mashhad, Iran; 4 Department of Biostatistics, School of Health, Mashhad University of Medical Sciences, Mashhad, Iran; 5 Social Determinants of Health Research Center, Mashhad University of Medical Sciences, Mashhad, Iran

**Keywords:** Caregiver, Mother, Motivation, Intellectual and developmental disabilities, Autism spectrum disorder, Cerebral palsy, Down syndrome, Children, Self-determination theory, Qualitative study

## Abstract

**Background:**

The motivation of caregivers plays a crucial role in the treatment, follow-up, and care of children with intellectual and developmental disabilities. Previous studies have focused on the older people and end-stage diseases, while giving less attention to the motivation of mothers caring for children with special needs. This study aimed to explore the motivations of mothers caring for children with intellectual and developmental disabilities in Iran.

**Methods:**

This study employed a qualitative approach, guided by the Self-Determination Theory. Purposeful sampling was initially used, followed by theoretical sampling until data saturation was achieved. Data were collected through semi-structured interviews with 26 mothers of children with intellectual and developmental disabilities. Mayring’s seven-step directed content analysis approach was utilized for coding and categorization. The research adhered to ethical standards and ensured data trustworthiness through credibility, dependability, confirmability, and transferability measures.

**Results:**

The findings revealed that mothers’ caregiving motivations could be classified into four main categories: (I) intrinsic, (II) identified-extrinsic, (III) introjected-extrinsic, and (IV) external-extrinsic. Additionally, twelve sub-categories were identified within these four main categories.

**Conclusion:**

The findings revealed that mothers demonstrated varying levels of intrinsic and extrinsic motivations in caring for children with intellectual and developmental disabilities. By recognizing and enhancing the diverse sources of motivation, healthcare providers and policymakers can better support mothers in their caregiving roles, ultimately contributing to improved outcomes for both the mothers and their children.

**Supplementary Information:**

The online version contains supplementary material available at 10.1186/s12887-024-04957-y.

## Background

Intellectual and developmental disabilities (IDDs) are increasingly prevalent in various countries [1]. IDDs refer to a condition that leads to significant limitations in mental performance, such as reasoning, learning, problem-solving, and adaptive behaviors including social and functional skills that are identified before the age of 18 [2, 3]. Based on these definitions, a wide range of disorders such as autism, epilepsy, cerebral palsy (CP), Down syndrome, fetal alcohol syndrome, and other conditions that result in developmental challenges and intellectual impairment can be classified under IDDs [2]. According to the results of previous studies, the prevalence of disability in Iran is around 1.3% [4].

Children with IDDs can experience various physical and psychological complications and require care from others, typically family members, and relatives known as informal or family caregivers [5]. In Iran, over 94% of caregivers for children with CP are mothers [6]. As time passes, the demand for care from family caregivers grows, highlighting their need for support and assistance from those around them [7]. Research indicates that caregivers may encounter behavioral, physical, and psychological issues due to the burden of caregiving [8]. This burden can also impact caregivers’ motivation for providing care.

The motivation of caregivers plays a vital role in the treatment and care of children. Previous studies have demonstrated a direct correlation between caregivers’ motivation and children’s treatment follow up [9]. A crucial aspect of caring for children with IDDs involves carrying out rehabilitation to achieve effective progress and recovery. Identifying both intrinsic and extrinsic motivations related to rehabilitation is essential to attain the goal of care [10]. Understanding the motivation of family caregivers to initiate and maintain their caregiving responsibilities is crucial, yet not fully comprehended.

Our search in databases revealed that the majority of studies concentrate on the motivation of caregivers of older people patients, while only a few studies have examined the motivation of caregivers in the realm of child care [11, 12]. In a qualitative study, the experiences and motivations of caregivers looking after dementia patients at home were categorized into three categories: prioritizing the parents’ needs to receive medical care above their own, expressing appreciation and thankfulness towards the elders through respect and involvement in their treatment, and acknowledging the parents’ capability to lead a better life [11].

The most significant theory on motivation is associated with Self-Determination Theory (SDT) [13]. According to this theory, individuals exhibit various levels and forms of motivation, ranging from amotivation or lack of self-determination to intrinsic motivation, which is entirely self-driven [14]. Extrinsic motivation falls between amotivation and intrinsic motivation and comprises the following dimensions: (I) Externally regulated, where an individual has minimal control and autonomy, performing a behavior solely for external rewards or to avoid punishment. (II) Introjected regulation, is where the individual holds some control over the activity, engaging in the behavior to alleviate guilt, and anxiety, or to maintain pride, demonstrate ability, and preserve a sense of self-worth. (III) Identified regulation, indicating a conscious acceptance and valuation of a goal from the individual’s perspective. It holds more strength than the preceding types, providing the individual with greater autonomy. (IV) Integrated regulation, is completely controlled by the individual and aimed at achieving distinct goals without deriving pleasure internally. Volunteerism plays a pivotal role in the integrated type [14]. In the current study, we considered the last two regulators as constituting a single dimension.

Maintaining motivation to care for the family caregiver, typically the mother of a child with IDDs who has to provide long-term care, is crucial. Past studies have demonstrated that factors such as the perceived burden of care, the nature of the relationship between the caregiver and the care recipient, available resources, and adaptation mechanisms can impact the motivation of caregivers of older people [7]. However, the motivation of mothers caring for children with IDDs has not been extensively explored. Therefore, identifying the motivations of mothers caring for children with IDDs through a qualitative study can serve as a foundation for future research aimed at understanding the factors influencing caregiving motivation and implementing appropriate interventions to maintain and enhance the motivation of these caregiving mothers. Consequently, the present study was performed to explore the motivations of mothers caring for children with IDDs in Iran.

## Methodology and methods

### Study design and theoretical framework

This study was a qualitative directed content analysis. Qualitative research is an approach to exploring and understanding the meaning among individuals facing a particular issue. Simply put, the goal of qualitative research is to uncover the significance of an event among those directly involved [15]. One common method employed in qualitative research is content analysis. Content analysis seeks to organize and derive meaning from gathered information, producing tangible insights. Content analysis is typically undertaken through three approaches: conventional, directed, and summative. The conventional approach is utilized when limited information is available on a particular topic. By contrast, the directed approach involves establishing categories based on existing conceptual models or theories, followed by matching the extracted codes to these categories [16].

In qualitative content analysis, various methods have been introduced, including Graneheim and Lundman, Hsieh and Shannon, Elo and Kingas, and others, each with its own steps [17–19]. Another method of content analysis is associated with Mayring, which has been specifically designed for conventional and directed approaches [16, 20]. Given the existence of a well-developed theory like SDT in the field of motivation, in the current study, we utilized Mayring’s directed content analysis approach to elucidate the understanding and experiences of mothers with children with IDDs regarding the extrinsic and intrinsic motivations of caregiving based on SDT principles.

### Participants and settings

After obtaining approval from Research and Ethics committees of Mashhad University of Medical Sciences to conduct sampling at the Children’s Neurology Department and Clinic of Qaim Mashhad Hospital, the Mashhad Autistic Children’s Centers, and the Esfarayen Educational and Rehabilitation Center for children with special needs, the study proceeded. The participants in this study were mothers of children with autism, CP, and Down syndrome with IDDs who possessed extensive experience in caring for IDDs children, were willing to share their experiences with the researchers, and consented to having the interviews recorded.

The mothers in this study ranged in age from 30 to 55 years, and their children’s ages ranged from one year and seven months to eighteen years. One mother had twin children with autism, while another mother, besides caring for a child with CP, had previously cared for another child with the same condition. Eleven mothers cared for children with CP, nine for children with autism, and six for children with Down syndrome (Table 1).

**Table 1:** Information of included caregivers

Participants	Mother’s age (year)	Education status	Child’s age	Occupational status	Child’s diagnosis
P1	44	Bachelor of science	7	Teacher	CP
P2	32	Diploma	11	Housewife	Down Syndrome
P3	45	Primary education	4.5	Housewife	CP
P4	55	Middle school	12	Housewife	CP
P5	42	Primary education	5	Housewife	CP
P6	39	Middle school	6	Tailor	Autism
P7	40	Middle school	8	Housewife	Autism
P8	52	Primary education	14	Housewife	Down Syndrome
P9	47	Diploma	10	Housewife	Down Syndrome
P10	33	Primary education	5.5	Farmer	CP
P11	55	Ph.D.	17	University faculty member	CP
P12	38	Primary education	17	Housewife	Down Syndrome
P13	32	Middle school	6	Housewife	Autism
P14	33	Bachelor of science	6.5	Housewife	Autism
P15	31	Primary education	7	Housewife	Autism
P16	40	Primary education	7	Tailor	CP
P17	35	Master of science	1.5	Academic Advisor	CP
P18	40	Diploma	15	Housewife	Autism
P19	36	Primary education	6	Housewife	Autism
P20	50	Primary education	18	Housewife	CP
P21	30	Diploma	10	Tailor	Down Syndrome
P22	35	Diploma	7	Housewife	Autism
P23	38	Middle school	8	Housewife	Autism
P24	50	Middle school	9	Housewife	CP
P25	41	Diploma	13	Housewife	Down Syndrome
P26	36	Middle school	13	Tailor	CP

### Data collection

In the current study, semi-structured face-to-face interviews were conducted with mothers of children with IDDs to explore the dimensions of care motivation. Sampling was initially purposeful and then theoretical, with efforts made during interviews to ensure necessary diversity in the sample. In this study, it was imperative to maintain an appropriate balance of mothers with children diagnosed with autism, CP, and Down syndrome in terms of interview sequence and frequency. Furthermore, careful consideration was given to the selection of mothers based on their caregiving experiences, employment status, and age to ensure the requisite diversity among participants. The first author carried out all interviews under the supervision of the research team.

Prior to each interview, the selected mothers were informed about the study details and its purpose, and written consent was obtained from them. The interview sessions began by collecting demographic information such as the mothers’ age, employment status, length of caregiving, and their child’s diagnosis.

The interview guide was used to conduct each session (Additional File 1). The interviews started with a general question: “Please describe the process of diagnosing your child’s disability.” Subsequently, specific questions were posed, such as: “Please share the motivations behind caring for your child.” Since this study employed directed content analysis based on SDT, questions were structured around the various dimensions of SDT. During each interview, voice recordings were made with the mothers’ consent using the voice recording software on a mobile phone. Subsequently, all recorded conversations were transcribed verbatim. Non-verbal cues, including changes in tone, emphasis, and pauses, observed by the researcher during the interviews, were also documented to gain a deeper understanding of participants’ emotional and psychological states, offering richer insights into their motivations and experiences [21]. For instance, a mother expressing interest and dependence on a disabled child while crying confirmed her verbal explanations.The interviews ranged in duration from 30 to 86 min. To enrich the categories and clarify certain ambiguities, the first, second, and sixth interviews were repeated. Each interview was recorded for accuracy. Interviews continued until data saturation, ensuring that no new categories or data emerged during subsequent interviews.

### Data analysis

**Fig. 1:**
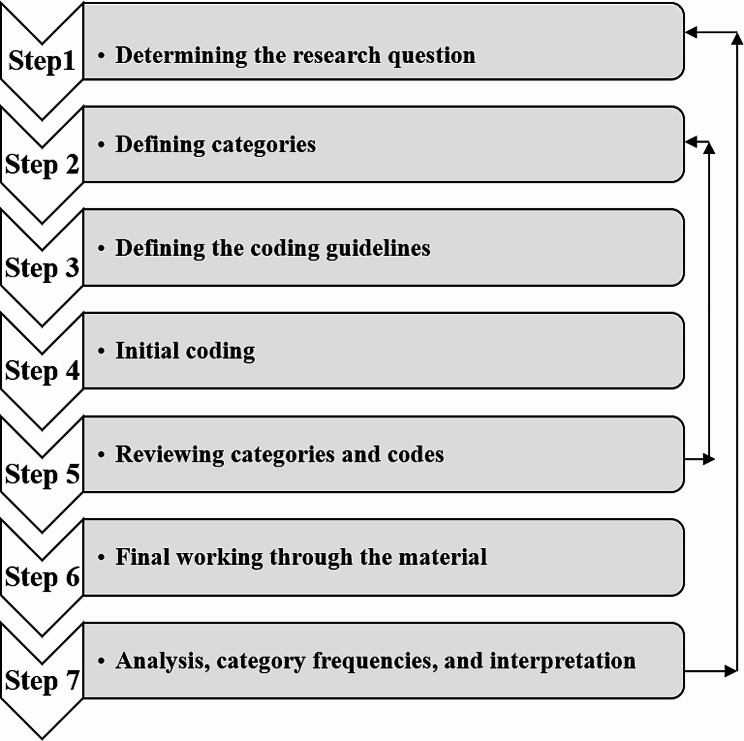
Steps of Mayring directed content analysis

In current research, data analysis was done simultaneously with data collection to refine data collection strategies and enhance the depth of understanding by immediately identifying categories and patterns [22, 23]. Also, the data management was carried out using MAXQDA (2020) software. Mayring’s directed approach was employed during the content analysis process. This approach consists of seven key steps (Fig. 1) [16]: (I) Determining the research question: “In this study, what are the dimensions of motivation in caring for mothers of children with IDDs based on SDT theory?” (II) Defining care motivation categories, including: intrinsic motivation, extrinsic-identified motivation, extrinsic-introjected motivation, and extrinsic-external motivation. (III) Defining the coding guidelines: a table with four columns (Variable and value, Definition, Anchor Samples, and Coding Rules) was prepared following Mayring’s recommended format, as outlined in Table 2. In the current study, we classified codes related to the mother-child relationship, and mother’s internal positive feelings, such as pleasure and interest, as part of the intrinsic motivation category. Codes linked to the mother’s goal, specifically the aim to achieve the progress and recovery of the child, and which were largely under her control, were categorized as identified-extrinsic motivation. Additionally, codes associated with behaviors aimed at alleviating guilt, fear, and anxiety, as well as, demonstrating ability, attaining internal rewards, and preserving a sense of self-worth, were classified under introjected-extrinsic motivation. On the other hand, external-extrinsic motivation included codes related to the mother’s environment, largely beyond her control, which could either encourage or coerce her to provide care. (IV) Initial coding: The text was coded systematically, starting from the beginning and aligning with the classification guideline. (V) Reviewing categories and codes: Continuous coding and category extraction occurred after each interview, with a thorough review of code placement following the initial coding of 20% of the data. Coding was carried out by the first author (SJH) after each interview, and the coded interview text and categories were then shared with the second author (MR) and other co-researchers (FA, JJ). In case of any disagreements about the coding, the team engaged in rigorous scientific discussions to resolve the discrepancies. (VI) Final working through the material: After ensuring stability in the coding process, the entirety of the coded text was categorized according to SDT [16]. (VII) In the final step, analysis, category frequencies, and interpretation of contingencies were conducted under the guidance of the research team, which includes a Ph.D. nursing student, a pediatric neurologist, a statistician, and a Ph.D. Nursing supervisor.

**Table 2:** Coding guidelines according to mayring directed content analysis

Variable	Definition based on SDT	Anchor Samples	Coding Rules
Intrinsic motivation	performing activities based on pleasure and interest.	“This child is mine, and I am his mother. Whether he is healthy or sick, I love him and take care of him.”	The codes pertaining to a mother’s care in terms of interest, pleasure, and as well as mother-child relationship.
Identified- Extrinsic motivation	Voluntarily carrying out behavior aligned with a personal reason that is within individual control, but lacks intrinsic pleasure.	“My top priority at the moment is my child’s development. There will be time for other goals later.”	Codes related to prioritizing care, engaging in rehabilitation both at home and in external settings, regulating care goals, and fostering hope for recovery.
Introjected- Extrinsic motivation	Engaging in activities based on internal rewards and punishments (performing the behavior to alleviate guilt, fear, and anxiety, and to maintain pride, demonstrate ability, prevent failure, attain internal rewards, and preserve a sense of self-worth.)	“I feel partly responsible for my child’s disability. Now, I must compensate for it by taking proper care.” “I fear that God will punish me if I neglect my child’s needs.”	Codes related to ensuring the mother’s capability to provide appropriate care, enhancing her sense of self-worth, and addressing feelings such as fear, guilt, and anxiety have positively influenced her motivation.
External- Extrinsic motivation	Engaging in activities based on external rewards and punishments over which the individual has no control.	“My wife’s encouragement to attend occupational therapy sessions at private centers has enhanced my motivation.”	Codes pertaining to the positive influence of family, relatives, healthcare providers, and government institutions on caregiving.

### Trustworthiness

Data trustworthiness was ensured using Lincoln and Guba’s criteria, which encompass credibility, dependability, confirmability, and transferability [24]. To establish credibility, the researcher engaged extensively in the analysis of interviews over six months, and the research team actively participated in the coding process. Peer debriefing was conducted with individuals familiar with qualitative research coding. The codes were also confirmed by the participants. For dependability, an external supervisor meticulously assessed the data and documentation in a step-by-step manner. Confirmability was reinforced by recording all activities from the initial stage of text search through coding and data extraction, which were then shared with external reviewers for validation. Lastly, to enhance transferability, participant characteristics, data collection methods, and analysis processes were meticulously detailed within the study [24, 25].

### Ethical considerations

Mashhad University of Medical Sciences ethical committee approved the study (Ethical code: IR.MUMS.NURSE.REC.1401.102). All methods were carried out in accordance with relevant guidelines and regulations. Written informed consent was obtained from all participants. Prior to their participation, mothers were provided with comprehensive information about the study’s objectives, design, and the guarantee of confidentiality. It was emphasized that participation was voluntary and that they could withdraw their consent at any time without providing a reason. Furthermore, the importance of maintaining anonymity and confidentiality was underscored. Interview data were anonymized during transcription, and the recorded interviews were stored in a strictly confidential manner.

## Results

In total, twenty-nine interviews were conducted with twenty-six mothers of children with IDDs. The mean ages of the mothers and children were 40.34 ± 7.44 years and 9.38 ± 4.33 years, respectively. The majority of the mothers (61.54%) had an education level below a high school diploma. Also, most mothers (69.23%) in the study identified as housewives (Table 2).

Upon data analysis, codes and subcategories were classified into four main categories: Intrinsic Motivation, Identified Extrinsic Motivation, Introjected Extrinsic Motivation, and External Extrinsic Motivation. (Table 3).

**Table 3:** Main categories and sub-categories of mothers’ motivation of caring a child with IDDs

Main category	Sub- category
Intrinsic motivation	Interest and dependence on the Child
	Feeling pleasure in caring for the child
	Innate maternal instinct
Identified- Extrinsic motivation	Achieving care goals
	Prioritizing childcare and rehabilitation over other responsibilities
	Having hope and confidence in the child’s recovery
Introjected- Extrinsic motivation	Ensuring optimal home care for the child
	Gaining a sense of self-worth through child progress
	Feelings of fear and guilt driving care
External- Extrinsic motivation	Providing proper care with the support of family
	Providing proper care with the social support
	Providing proper care with the support of official sources

### Intrinsic motivation

According to SDT, intrinsic motivation drives individuals to engage in activities for the sake of personal enjoyment and interest [14]. The findings of this study suggest mothers are motivated by their inherent interest in and emotional dependence to their children. They derive pleasure from caregiving and are guided by their innate maternal instincts.

The mothers in the current study expressed that their caregiving was driven by their interest in and dependence on their child with IDDs *P17 “I am highly dependent on my child and feel that my life has meaning and purpose only through him. I cannot contemplate being away from or losing my child.”* Caregivers prioritize looking after their child over other family members and even themselves, unable to bear seeing their child in discomfort. *P5 “I suffer a lumbar disc hernia*,* but I still lift and hold my daughter myself. She doesn’t need a wheelchair or a stroller. I manage all her needs by myself.”* Mothers also reported that their interest in their child increased over the years of caregiving. *P5 “Over the years*,* I have consistently provided care for my disabled child. This indicates that my care and interest in him have not only remained persistent but have also increased compared to the past.”*

In addition to their interest with their child, the mothers derive pleasure from providing care, amusement, and happiness to their child. *P1 “It’s a fulfilling sensation when*,* as a mother*,* I can feed my child*,* dress him*,* and attend to his needs.”* Also, *P16* added: *“I derive significant personal pleasure from spending time with my child. During my travels*,* I ensure that my child accompanies me. His presence is a constant source of companionship*,* and I prefer to personally attend to his care rather than delegating this responsibility to others.”*

Furthermore, participants indicated that their motivation for caregiving arises from an intrinsic maternal instinct, which acts as a powerful impetus deeply embedded in the mother-child relationship. This instinct drives mothers to meet their caregiving responsibilities, irrespective of the child’s health status. *P3 “It’s mostly my maternal instinct that propels me to care for my child. I may feel exhausted at times*,* but I persevere because I am his mother.” P5* added, *“Because he is my child*,* no matter how ill he may be*,* I must look after him and attend to his needs.”*

### Identified- extrinsic motivation

In the context of SDT, identified regulation represents a form of extrinsic motivation, where activities are pursued to achieve specific goals and are deemed personally significant [14]. Individuals with this type of motivation may not derive intrinsic pleasure or interest from the activity itself. The findings of this study suggest mothers are motivated by the achievement of caregiving goals, the prioritization of childcare and rehabilitation, and the maintenance of hope and confidence in their child’s recovery.

Within this category, mothers strive towards a broad goal of seeing progress and development in their child with special needs. Additionally, they set specific objectives for themselves, such as helping their child lead a more typical life, enhancing learning and educational opportunities, improving speech, facilitating social integration, and fostering independence. *P6 “I wish for my child to be able to attend school and communicate like my other two children.”* To work towards these objectives, mothers engage in rehabilitation efforts both at home and outside, and seek specialized treatments. *P13 “Every day*,* I dedicate two hours to occupational therapy for my child*,* under the remote guidance of a specialist from Tehran through video calls.”*

Progress in the child’s development is facilitated by the mother’s ability to provide optimal care at home. To enhance their caregiving, mothers seek knowledge relevant to their child’s condition from healthcare professionals, peers facing similar challenges, staff at rehabilitation centers, online forums, television resources, and their own experiences. *P6 " I participate in virtual support groups on various platforms where mothers exchange their experiences and strategies. They share the measures they use for their child’s developmental progress*,* and I attempt to apply their advice.”*

The pursuit of progress and recovery demands significant dedication from caregivers, often leading mothers to prioritize caring for their children with IDDs over personal ambitions, social engagements, and even the needs of other family members. *P1 “I hardly find time for unnecessary activities. I often remind myself to focus on my child’s rehabilitation. Later on*,* if possible*,* I can pursue my own interests.”* Also, *P8 “Although I was invited to a relative’s birthday party*,* I chose to take my son to the rehabilitation center instead*,* as his therapy is crucial.”* The rehabilitation of a disabled child is of paramount importance to a mother, often leading her to prioritize and facilitate this process at home. *P6:“We have dedicated a room in our house specifically for our child’s rehabilitation. We have purchased the necessary equipment and are actively engaging in rehabilitation activities with our child at home.”*

Also, on the journey towards achieving their objectives, mothers of children with IDDs often harbor hopes for their child’s progress and recovery, and in some cases, they are even confident in the potential for significant improvement. *P14 “I firmly believe that my child will eventually show improvement; that certainty keeps me going.”* While not all mothers express such unwavering confidence, many remain hopeful. *P18 “I still hold onto hope that my child will learn to communicate with at least four words*,* conveying his thoughts to othe*rs.**”**

### Introjected- extrinsic motivation

Within the framework of SDT, introjected regulation reflects a form of extrinsic motivation in which individuals engage in activities based on internal values, such as self-imposed rewards or punishments, with a moderate degree of personal control [14]. The findings of this study suggest mothers’ motivation to care for their children with special needs is driven by a commitment to ensuring optimal home care for their child, gaining a sense of self-worth through child progress, and feelings of fear and guilt.

The mother’s caregiving is influenced by her abilities and confidence in managing her child’s needs at home. The mother believes she possesses the necessary physical capabilities, adequate knowledge and skills, and dedicates the appropriate time to ensure the child’s various needs are met, to manage the child’s involuntary behaviors, and to provide suitable physical care. *P6 “I am always present at home and not employed*,* so I must look after my child myself.”* Also, *P16 “Over the past eight years*,* I have diligently prevented bedsores in my child by regularly applying vitamin A + D ointment*,* massaging him*,* and changing his position every two hours*,* even during the night.”*

Furthermore, the mother views caregiving as valuable, recognizing its contribution to the child’s developmental progress. Even small advancements are significant to her. *P21 “It was extremely challenging initially*,* but as soon as he began talking and walking*,* it became incredibly rewarding for me.”* Also, *P6* added *“Seeing that the care and follow-ups have an effect is very valuable to me. For example*,* my child has learned a new word*,* or his behavior has changed and progressed*,* making me realize that this child is growing.”*

Additionally, the mother’s caregiving is driven by fears and concerns about potential regression in the child’s development, the child’s vulnerability to injuries and trauma, and a desire to avoid divine retribution. *P22 “I fear that neglecting proper care will lead to negative consequences for us. I fear God.”* Also, *P8 “I constantly remind my husband of the importance of caring for our child to prevent his condition from deteriorating further. The older he gets*,* the harder it becomes to treat him.”* The mother also tends to care for the child to alleviate feelings of guilt stemming from perceived past shortcomings. *P5 “I blame myself for not breastfeeding my child adequately during infancy*,* thinking it hindered his brain development. That’s why I’m vigilant and seek treatment at various medical facilities.”* Moreover, she strives to avert potential remorse by providing diligent care to prevent any negative outcomes affecting her child. *P5 “I care for my child so that I won’t harbor regrets if something unfortunate happens due to my lack of attentiveness.”*

### External- extrinsic motivation

External regulation, as suggested by SDT, pertains to extrinsic motivation driven by external rewards or punishments over which individuals have little or no control [14]. The findings of current study suggest mothers are motivated by the support they receive from family, social networks, and official resources, which enables them to provide proper care for their children.

The provision of care can be significantly influenced by the support and assistance of family members, such as a spouse and healthy children. These family members offer verbal, physical, and financial support, aiding the mother in properly caring for the child. They also create a supportive atmosphere, prioritizing the well-being and progress of the child with disabilities. *P7 “One of my motivations is my healthy and older daughter*,* who wishes for her sisters to be as well as her and to have companionship.”* Also, *P16*,* “The environment at home is such that everyone pitches in to help care for my disabled daughter*,* and we support each other*,* which prevents me from feeling fatigued. I’m grateful for the support from my family*,* especially my wife.”*

Moreover, social support, including encouragement and acceptance of the child with IDDs by neighbors and relatives, as well as the sharing of effective experiences from other mothers in similar situations, can motivate the mother to provide appropriate care for the child. *P16 “My parents closely monitor the condition of my disabled child and consistently recommend taking the child to rehabilitation centers and doing exercises with him at home.”* Also, *P13 “We mothers at the rehabilitation center empathize with each other. We share our experiences and feelings because we all encounter similar challenges*,* offering mutual understanding and motivation.”*

In addition to the support of family members and the community, the mother’s acknowledgment from medical professionals and government organizations plays a crucial role in enabling her to provide suitable care. *P5 “The female doctor at the ICU praised my caregiving efforts*,* giving me a great sense of satisfaction. She commended me for my dedication and now I strive to enhance my caregiving even further.”* Another mother, *P21*,* “The welfare center now offers transportation services for my son to the rehabilitation center*,* and all the facilities at the center are provided to us free of charge. This support motivates me to continue my child’s rehabilitation journey.”*

## Discussion

The present study aimed to explore the motivation of mothers with children with IDDs in Iran. The findings revealed that mothers’ caregiving motivation in Iran could be categorized into four main categories: Intrinsic motivation, Identified-Extrinsic motivation, Introjected-Extrinsic motivation, and External-Extrinsic motivation. The following discussion is structured around each of the extracted categories.

### Intrinsic motivation

Mothers driven by intrinsic motivation are not influenced by external influences and independently provide care out of genuine interest and pleasure [14]. In this study, mothers attended to their children with IDDs fueled by a sense of genuine interest, feeling pleasure, and an innate maternal instinct. Behaviors guided by intrinsic motivation tend to be sustainable as individuals engage in activities that bring them personal satisfaction [26]. Prior studies have predominantly focused on the intrinsic motivation of family caregivers assisting adults with chronic conditions like cancer, Parkinson’s, and dementia, highlighting the significant role of strong commitment, love, loyalty, and respect as primary drivers for their caregiving efforts [7, 27, 28]. Studies focusing on caregiving for children highlight the deep love and attachment between mothers and their children. Mothers spare no effort in ensuring the well-being of their children, often bearing a significant caregiving burden that can lead to increased distress [29]. Similarly, caregivers of individuals with advanced cancer emphasized the parent-child bond and affection as primary motivators for their caregiving involvement, consistent with findings of current research [30]. Also, the presence of the children with IDDs beside the mother evokes a sense of pleasure and motivates her to persist in providing care for her child, despite the challenges associated with caring for a child with IDDs. This sense intensifies significantly when accompanied by the child’s progress [31].

### Identified-extrinsic motivation

This category comprised three sub-categories: achievement of caregiving goals, prioritization of childcare, and maintenance of hope and confidence in their child’s recovery. Within the context of Identified-Extrinsic motivation, mothers establish specific goals for their children, striving for progress and expeditious recovery. Mothers believe that early treatment follow-ups increase the chances of attaining these goals. Establishing an attainable and accessible goal not only fosters maternal motivation for caregiving but also mitigates anxiety and depression. Nevertheless, setting goals without accounting for factors such as the child’s disability severity and mother’s capabilities may lead to adverse effects on her motivation [32].

Another sub-category involves the prioritization of their child’s care and rehabilitation over other responsibilities, as mothers perceive a substantial impact on their lives due to their children with IDDs. This compels them to navigate challenging situations for the well-being of both themselves and their children, leading to a prioritization of their child’s treatment and rehabilitation over other responsibilities. For instance, even an employed mother sets aside her work to focus on caring for her child, managing medical appointments, and rehabilitation program. This issue was highlighted in a study involving caregivers of individuals with Down syndrome, where mothers opted to leave their jobs to care for their children [33].

Another significant finding in this category was the mothers’ hope and confidence in their child’s recovery. Hope is perceived by mothers as the anticipation of positive outcomes, such as progress and recovery for their children with IDDs in the future. This sense of hope plays a crucial role in motivating mothers to provide care for these children. The mothers in this study exhibited hopeful attitudes, sometimes even expressing confidence in their child’s recovery. Consequently, hope for recovery emerges as a critical factor motivating mothers to engage in rehabilitation and caregiving activities. Hope empowers mothers and reduces psychological distress [34]. The caregiving experience of a mother with a child with trisomy 18 demonstrates that hope significantly alleviates anxiety and depression [35]. Additionally, hope for recovery not only influences caregivers’ provision of appropriate care but also protects them from exhaustion and burnout, as observed in caregivers of patients with end-stage cancer [36].

### Introjected-extrinsic motivation

In the category of introjected-extrinsic motivation, a mother’s drive is influenced by her ability to provide optimal care at home, gaining a sense of self-worth through child progress, as well as experiencing emotions like guilt and fear. Mother’s ability to provide optimal care encompasses her physical and mental well-being, availability of time, absence of employment, and possession of adequate knowledge and skills to care for a child with special needs. Previous research has consistently shown that the caregiver’s poor health status acts as a hindrance to delivering appropriate care to the care recipient [37]. Additionally, if financial constraints are absent, a mother’s employment status can be perceived as a barrier to caring for a disabled child as it often limits the time available for caregiving. Also, studies aligned with the current research have indicated that a lack of necessary expertise in managing a care recipient’s condition can lead to an increased burden of caregiving, subsequently negatively impacting the motivation to provide care [38].

In the current study, some mothers reported that observing progress in their child’s developmental status encourages a sense of self-worth and internal reward, motivating them to invest more care and effort. Even subtle improvements in their child’s developmental status validate their caregiving efforts, reinforcing their dedication. Thus, the child’s progress serves as a significant motivating factor for these mothers. This subcategory has been comparatively underexplored in previous studies.

Other subcategory, identified as ‘Feelings of fear and guilt as motivators for caregiving,’ represents a significant aspect of Introjected-Extrinsic motivation within the study. Certain mothers attributed their child’s disability to perceived lapses in prenatal or postnatal care, leading to feelings of guilt that compel them to compensate through caregiving. This phenomenon mirrors observations seen in caregivers of cancer patients, where similar experiences of guilt motivate caregiving behaviors. It’s important to note that heightened guilt can precipitate adverse psychological outcomes, including depression and anxiety, among caregivers [39].

In this study, fear stemming from three distinct sources emerges as a significant motivator for caregiving among mothers of disabled children. Firstly, mothers express a fear that neglecting care and treatment follow-up could result in their child’s regression. Secondly, concerns about inadvertently harming the child, such as through agitation or aggression arising from environmental factors, drive mothers to provide attentive care. Lastly, a fear of divine retribution motivates mothers, as they believe their actions toward the disabled child are observed by a higher power, and inadequate care may lead to adverse consequences in other areas of their lives. Notably, fear is commonly observed among caregivers of terminally ill patients, such as those with cancer, contributing to their emotional distress [40].

### External-extrinsic motivation

Based on the findings of this study, three subcategories of external-extrinsic motivation were identified: providing proper care with family support, providing proper care with social support, and providing proper care with support from official sources. Mothers in the current study highlighted the significant role of their spouse and healthy children in motivating them to care for a disabled child. Family support manifested through verbal encouragement, financial assistance, and help with caregiving. It is crucial for these mothers that their families accept the presence of a children with special needs and prioritize the child’s progress and recovery. Such support is regarded as an external reward for the mothers. The significance of family support has been highlighted in other studies as well, where caregivers of patients at the end of life have also emphasized this aspect [41]. Apart from family support, mothers also reported that encouragement from relatives, neighbors, and other mothers of children with IDDs plays a vital role in motivating them to provide care. This encouragement includes positive feedback on the disabled child’s progress, effective communication, and acceptance. Recent studies have revealed that caregivers of children with Down syndrome particularly value societal acceptance and positive reactions towards their child [33].

The third subcategory relates to receiving support from official sources, which effectively creates motivation and facilitates proper care for a child with special needs. Mothers frequently refer to treatment centers such as hospitals, pediatric neurology clinics, and rehabilitation centers for services including physiotherapy, occupational therapy, and speech therapy based on their children’s specific needs. Support from medical staff plays a key role in motivating mothers in various ways. Proper advice and training enhance the mothers’ knowledge and skills in providing appropriate care. Additionally, encouragement and positive feedback regarding the child’s progress from healthcare providers serve as significant incentives. A study focusing on children with CP indicated that encouragement from treatment staff to engage in their child’s rehabilitation positively influenced maternal motivation [40]. Furthermore, financial support from government institutions, such as the Welfare Organization, which is the primary supporter of children with special needs in Iran, alleviates the financial burden associated with caregiving, allowing mothers to focus more on the quality of care. By establishing rehabilitation centers, this organization also significantly contributes to the progress and recovery of these children. For mothers, the availability of numerous rehabilitation centers and the facilitation of transport for children from home to these centers are essential factors in supporting their caregiving efforts. A similar study highlighted caregivers of children with Down syndrome expressing satisfaction with access to treatment facilities such as hospitals and specialists [31]. However, it is crucial to note that many children face physical ailments such as orthopedic issues, heart diseases, dental problems, which impose a significant financial burden on their families. Consequently, families expect greater financial support from government institutions for the treatment of children with IDDs [42].

Based on the results of the present study, we offer both general recommendations and specific suggestions for Iran. Our findings highlight the need to consider the motivation of mothers caring for children with disabilities in a multifaceted manner. These mothers require verbal, physical, and financial support from family, society, and government to effectively care for their children. Additionally, healthcare providers at treatment and rehabilitation centers play a crucial role in motivating mothers by providing essential knowledge and skills, offering interventions to foster hope, and setting realistic goals. In the context of Iran, children with special needs and their families receive support from the welfare organization. Given that our study was conducted in Iran, these findings are particularly relevant for the managers of this organization. Enhancing the motivation of mothers to provide care can reduce their dependency on the welfare organization. This shift can encourage mothers to care for their children at home rather than relying on welfare boarding centers. Financial support and the availability of rehabilitation centers are critical in sustaining and enhancing the motivation of these mothers.

## Conclusion

The results of this study highlight the multifaceted nature of caregiving motivation among mothers of children with IDDs. Twelve distinct subcategories of motivation were identified and classified into four main categories: intrinsic, identified-extrinsic, introjected-extrinsic, and external-extrinsic. Intrinsic motivations included the mothers’ interest and dependence on their child, the pleasure derived from caregiving, and innate maternal instincts. Identified-extrinsic motivations involved achieving care goals, prioritizing childcare and rehabilitation, and maintaining hope and confidence in the child’s recovery. Introjected-extrinsic motivations were driven by the desire to ensure optimal home care, gain a sense of self-worth through the child’s progress, and feelings of fear and guilt. External-extrinsic motivations encompassed the support received from family, social networks, and official sources. Understanding these motivations can inform the development of targeted support programs and interventions that address the specific needs and challenges faced by these caregivers. By recognizing and enhancing the diverse sources of motivation, healthcare providers and policymakers can better support mothers in their caregiving roles, ultimately contributing to improved outcomes for both the mothers and their children.

### Implication of findings

This study aimed to identify the caregiving motivations of mothers with children who have IDDs and categorized these motivations as intrinsic and extrinsic, including identified, introjected, and external factors. According to SDT, a shift towards intrinsic motivators is associated with an increased likelihood of sustained and enhanced care for disabled children, as it affords greater autonomy to the mother in their caregiving role. In light of these findings, healthcare professionals, including nurses and physicians, are encouraged to adopt a holistic and multidisciplinary approach in supporting mothers of children with IDDs. Recognizing and respecting the diverse caregiving motivations of these mothers is essential for fostering effective care. By understanding the intrinsic motivations, healthcare professionals can strive to strengthen the emotional bond between mothers and their children, thereby promoting a nurturing and rewarding caregiving experience. Furthermore, insights into identified motivations can help guide healthcare professionals in establishing realistic care goals, providing resources to bolster hope and confidence in the child’s development, and assisting mothers in prioritizing childcare effectively. Addressing introjected motivations emphasizes the need for healthcare providers to offer practical guidance, emotional support, and specialized caregiving skills training to alleviate feelings of fear and guilt and enhance the mothers’ ability to provide appropriate care at home. In addition to these considerations, healthcare professionals can play a pivotal role in facilitating access to external sources of support, such as family, social networks, and official services, to fortify the caregiving efforts of mothers of children with IDDs.

For policymakers, extensive support for caregivers is paramount. This support should encompass the provision of facilities and interventions designed to enhance caregivers’ knowledge, mitigate negative emotions, nurture hope for progress and recovery, and ultimately cultivate a fulfilling caregiving experience rooted in intrinsic motivators.

### Study limitations

While efforts were made to enhance the transferability of the study results to other settings through diverse sampling and a thick description of the research process, application of the findings in alternative contexts is subject to limitations inherent in qualitative studies. Also, it is important to recognize that IDDs encompass a broad spectrum of disease. This study focused on three specific disorders: Down syndrome, autism, and CP. We sought to maximize diversity by recruiting participants representing each of these three conditions. Nevertheless, it should be noted that mothers’ experiences may vary based on the specific nature of the disorder. Additionally, socio-demographic factors, including income status and place of residence, may also influence maternal experiences.

### Electronic supplementary material

Below is the link to the electronic supplementary material.

Supplementary Material 1

## Data Availability

The datasets used and/or analyzed during the current study are available from the corresponding author upon reasonable request.

## Abbreviations

IDDs: Intellectual and developmental disabilities
SDT: Self- determination theory
CP: Cerebral palsy
