# Supplementary material for: Motivation in caregiving among mothers of children with intellectual and developmental disabilities in Iran: A qualitative study
Source: BMC Pediatr. 2024 Jul 25;24:472. doi: 10.1186/s12887-024-04957-y (PMC11271013; doi:10.1186/s12887-024-04957-y)
Supplement: Supplementary file 1 — Supplementary Material 1 [file 12887_2024_4957_MOESM1_ESM.docx]

**Guide to Qualitative Interviews**

In conducting the current study's interviews, three stages—preparation, main, and final—were carefully considered.

**1. Preparation Stage:**

During this stage, mothers were provided with comprehensive explanations about the research, its aims, the process of study approval, and the acquisition of ethical clearance. They were assured that the interview results would be published generally, and the participants' details would remain confidential with the researcher. To establish two-way communication, mothers were engaged with general questions about their child's illness progression. For instance, "Please explain the reason for visiting this center." Subsequently, "Please provide explanations about the process of diagnosing your child's illness." Following this, our focus shifted to the second stage, which centered on questions regarding the mothers' caregiving motivation.

**2. Main Stage:**

Given the guided approach in our study, the majority of questions were tailored to explore various aspects of the theory and the participants' narratives. Throughout the interviews, the interviewer focused on key concepts expressed by the mothers to gain a deeper understanding of their experiences.

Mothers were prompted to describe a typical day of caring for their disabled child, followed by an exploration of their motivation for caregiving. They were then asked to recount a day when they felt highly motivated to provide care.

Subsequent questions sought to delve into dimensions of motivation derived from the self-determination theory. Specifically, inquiries related to the external dimension of extrinsic motivation were pursued under the title: "Please explain the influence of the surrounding environment on your caregiving motivation." Participants were encouraged to expound on each condition and elucidate the impact of these factors on their motivation to care. For instance, "You mentioned the influence of your wife in our prior conversations—could you elaborate further on this?"

The interview then explored questions pertaining to the introjected-extrinsic motivation dimension. For instance, mothers were asked how their child's condition, requiring continuous care, made them feel, and to articulate the impact of these emotions on their motivation to care. Additionally, mothers were queried about the effects of fear and guilt on their caregiving motivation.

As for the dimension of identified-extrinsic motivation, mothers were asked to explain their perceived ability to care for their disabled child, whether they considered themselves the best person for the task, and to outline their caregiving goals. Subsequently, mothers were prompted to discuss intrinsic motivation, particularly the influence of positive emotions on their motivation to care for a child with special needs.

**3. Final Stage:**

In the third stage of the interview, mothers were encouraged to provide an explanation or raise questions about care motivation that had not been previously addressed. Finally, mothers were informed about the possibility of a follow-up interview or further contact to address any ambiguities.

**Exploratory Questions:**

Exploratory questions were interwoven among the foundational questions to elucidate and resolve any ambiguities in understanding the participants' experiences. These questions aimed to convey to the mothers that their perspectives were valued, encouraging them to express their experiences in greater detail. Examples of such questions include: "Please provide further insight into this matter" and "Could you elaborate on your earlier explanations by citing a specific experience?"
